# Trends in measures of handgrip strength from 2014 to 2017 among Korean adolescents using the Korean National Health and Nutrition Examination Survey Data

**DOI:** 10.1186/s13104-020-05112-3

**Published:** 2020-06-30

**Authors:** Yunkoo Kang, Jieun Kim, Seung Kim, Sowon Park, Hyunjung Lim, Hong Koh

**Affiliations:** 1 Department of Pediatrics, Yonsei University Wonju College of Medicine, Wonju, South Korea; 2 Department of Pediatrics, Severance Children’s Hospital, Yonsei University College of Medicine, 50-1 Yonsei-ro, Seodaemun-gu, Seoul, 03722 South Korea; 3 Research Institute of Medical Nutrition, Kyung Hee University, Seoul, 02447 South Korea; 4 Department of Medical Nutrition, Graduate School of East–West Medical Science, Kyung Hee University, Yongin, Gyeonggi-do 17104 South Korea

**Keywords:** Sarcopenia, Adolescent, Muscles, Handgrip Strength

## Abstract

**Object:**

Measuring handgrip strength is a useful method to evaluate sarcopenia. No study has shown the trends of handgrip strength to weight ratio among Korean adolescents by year. This study aimed to determine the trends of handgrip strength among Korean adolescents using data from the Korea National Health and Nutrition Examination Survey (KNHANES). Data of 2304 adolescents who participated in the KNHANES between 2014 and 2017 were obtained. The handgrip-to-weight ratios were categorized by age, sex, and year.

**Results:**

Handgrip strength in adolescents decreased from 28.67 kg in 2014 to 27 kg in 2017 (P for trend < 0.05). The handgrip-to-weight ratio also decreased from 51.48 in 2014 to 48.18 in 2017 (P for trend < 0.05). The handgrip strength and handgrip-to-weight ratio also decreased among boys and girls over the years 2014–2017 (P for trend < 0.05). The results of the present study indicate that the handgrip-to-weight ratio decreased in Korean adolescents from the years 2014 to 2017, and a declining overall ratio indicates a decrease in the health status among Korean adolescents. Hence, there is a need to review the health status of Korean adolescents.

## Introduction

Sarcopenia is defined as a progressive and generalized skeletal muscle disorder that involves the accelerated loss of muscle mass and function [1]. Furthermore, sarcopenia is known to be associated not only with aging but also with other systemic diseases, such as metabolic syndrome and non-alcoholic fatty liver disease (NAFLD) in adults [2, 3]. Thus, evaluating sarcopenia is important, for which there are many measurement tools available. Computed tomography, magnetic resonance imaging, and dual-energy X-ray absorptiometry can measure muscle mass. Additionally, handgrip strength and knee flexion/extension strength can be used to assess muscle strength [4, 5]. Handgrip strength assessment is a very simple and reliable method because it is correlated with several medical conditions [6, 7], even in adolescents and children [8–11]. Thus, handgrip strength has been used to identify sarcopenic obesity in children [12]. A recent study reported a strong relationship between handgrip strength and obesity and metabolic syndrome in adults and adolescents [13–17]. Steffl et al. showed that handgrip strength can help identify children at risk for sarcopenic obesity [12]. The handgrip strength represents the strength of the simple muscle force, however, it can also represent something more important. The decreases in grip strength can be attributed to problems with several medical conditions, such as children’s mental health, obesity and metabolic syndrome as mentioned above. Based on this, if we can figure out the trends of handgrip strength, medical condition of children can be evaluated easily. However, no study has shown the trends of handgrip strength to weight ratio among Korean adolescents by year. This study aimed to evaluate the trends of handgrip strength to weight ratio and estimate the overall health condition among Korean adolescents.

## Main text

### Methods

#### Data

The present study evaluated data from the 2014–2017 Korea National Health and Nutrition Examination Survey (KNHANES). Different participants were selected every year. These annual cross-sectional surveys are performed using multi-stage probability samples that are representative of the noninstitutionalized civilian Korean population. The data of selected participants has specific sampling weights factors because each data does not have equal probability of being selected. The data were analyzed after adjusting with specific sampling weights factors for each participant.

#### Subject selection

During 2014–2017, a total of 2988 individuals participated in the KNHANES. The present study included participants aged 10–18 years; participants with missing data were excluded. Thus, 2304 participants (1227 boys and 1077 girls) were included in the analysis. The participants were categorized by age and sex. The subjects were categorized into 9 age groups by each year, from ages 10 to 18 years.

#### Measurement of anthropometric and laboratory variables

Systolic blood pressure (SBP, mmHg) and diastolic blood pressure (DBP, mmHg) were measured to the nearest 2 mmHg using a mercury sphygmomanometer [Baumanometer Wall Unit 33(0850)]. Height was measured to the nearest 0.1 cm using a stadiometer (SECA 225, Seca GmbH & KG.), and weight was measured to the nearest 0.1 kg using a balance beam scale (GL-600-20, G-tech). Waist circumference was measured to the nearest 0.1 cm using a measuring tape (SECA 200, Seca GmbH & KG.). The body mass index (BMI) and waist-to-height ratio (WtHR) were calculated from the measured height and weight of participants (kg/m^2^ and waist (cm)/height (cm) × 100, respectively). Blood samples were collected from venous blood and levels of fasting glucose (mg/dL), aspartate aminotransferase (AST, U/L), and alanine aminotransferase (ALT, U/L), triglyceride (TG, mg/dL), cholesterol (mg/dL), high-density lipoprotein (HDL, mg/dL), were measured using a Hitachi Automatic Analyzer 7600 [18].

#### Handgrip strength measurement

The Takei digital grip strength dynamometer (Model T.K.K.5401, Takei Co., Ltd., Ishioka, Japan) was used to measure handgrip strength. The dynamometers were calibrated according to a standardized protocol and a special investigator checked the measurement to ensure it was done properly. Measurement of handgrip strength was performed after rest and light exercise. All participants, except those with a history of wrist surgery within 3 months or any wrist discomfort, underwent the handgrip strength test. The handgrip strength testing procedures was done according to the muscle strength procedures manual by Centers for Disease Control and Prevention [19, 20]. Handgrip strength was measured in a standing position with the arm and wrist in the anatomical position. Participants were asked to exhale and apply a maximal grip for 3 s, for a total of 3 repetitions each, starting with the dominant hand. The left and right hands were alternated. Sixty seconds of rest was allowed between each measurement. The highest handgrip strength value (in kg) between both hands was recorded and included in the analysis [12] (http://knhanes.cdc.go.kr/). Handgrip strength was calculated as a ratio (handgrip-to-weight, HGtW, (hand grip, HG/weight) × 100) which was used in the analysis [12, 15].

#### Statistical analysis

The SPSS software (version 23.0; IBM Inc., Armonk, NY) was used for statistical analyses of all data. Participants have different sampling probabilities of being selected, therefore each participant have proper weight factor. In order to represent the entire Korean adolescent population with small number of participants, data was adjusted with sampling weight factors. One-way analysis of variance was used to compare the mean value of the continuous variables of SBP, DBP, height, weight, waist, BMI, WHtR, glucose, cholesterol, TG AST, ALT. Continuous data was analyzed and expressed as mean ± standard error, applying weight factors. P for trend were calculated among years by linear regression with applying weight factors of survey design.

### Results

#### Characteristics of the participants from 2014 to 2017

The characteristics of the study participants from 2014 to 2017 are shown in Table 1. All values including age, SBP, DBP, height, weight, waist, BMI, glucose, TG, AST, ALT did not show any trends over the years 2014, 2015, 2016, and 2017. However, WtHR decreased and total cholesterol increased significantly between the years 2014 and 2017 (P for trend < 0.05) (Table 1).

**Table 1:** Participants characteristics in KNHANES 2014 to 2017

	Total (n = 2304)	2014 (n = 483)	2015 (n = 574)	2016 (n = 634)	2017 (n = 613)	*B* coefficient	*P* for trend
Age (year)	14.41 ± 0.06	14.28 ± 0.14	14.41 ± 0.13	14.47 ± 0.10	14.49 ± 0.14	0.067	0.272
SBP (mmHg)	108.54 ± 0.26	108.06 ± 0.61	108.90 ± 0.49	109.14 ± 0.53	108.05 ± 0.49	0.008	0.975
DBP (mmHg)	66.41 ± 0.22	65.70 ± 0.50	66.31 ± 0.40	66.94 ± 0.41	66.67 ± 0.47	0.349	0.098
Height (cm)	162.12 ± 0.28	161.69 ± 0.64	161.80 ± 0.53	162.32 ± 0.50	162.66 ± 0.50	0.343	0.180
Weight (kg)	56.08 ± 0.36	55.63 ± 0.86	57.14 ± 0.67	55.58 ± 0.68	55.96 ± 0.65	− 0.069	0.838
Waist (cm)	71.05 ± 0.26	70.47 ± 0.55	72.73 ± 0.49	70.91 ± 0.49	70.09 ± 0.51	− 0.319	0.188
BMI (z-score)	0.12 ± 0.32	0.10 ± 0.06	0.30 ± 0.68	0.01 ± 0.06	0.05 ± 0.06	0.117	0.115
WtHR	43.83 ± 0.14	43.55 ± 0.28	44.97 ± 0.28	43.69 ± 0.27	43.10 ± 0.28	− 0.280	0.028
Glucose (mg/dL)	91.85 ± 0.23	92.33 ± 0.67	91.88 ± 0.34	91.82 ± 0.36	91.37 ± 0.41	− 0.293	0.218
Cholesterol (mg/dL)	162.27 ± 0.66	157.52 ± 1.37	161.59 ± 1.23	164.01 ± 1.31	165.95 ± 1.35	2.755	< 0.001
TG (mg/dL)	86.08 ± 1.29	85.52 ± 2.95	88.29 ± 2.69	84.89 ± 2.26	85.61 ± 2.41	− 0.337	0.777
AST (U/L)	19.36 ± 0.23	18.87 ± 0.39	19.82 ± 0.66	19.14 ± 0.34	19.60 ± 0.40	0.143	0.438
ALT (U/L)	15.73 ± 0.46	14.87 ± 0.74	16.36 ± 1.22	15.55 ± 0.70	16.12 ± 0.94	0.284	0.465

Data presented as mean ± standard error with weighting of survey design

*SBP* systolic blood pressure, *DBP* diastolic blood pressure, *BMI* body mass index, *WtHR* waist to height ratio(waist(cm)/height(cm) × 100), *TG* triglyceride, *AST* aspartate aminotransferase, *ALT* alanine aminotransferase

#### Trends of handgrip strength among boys from 2014 to 2017

**Fig. 1:**
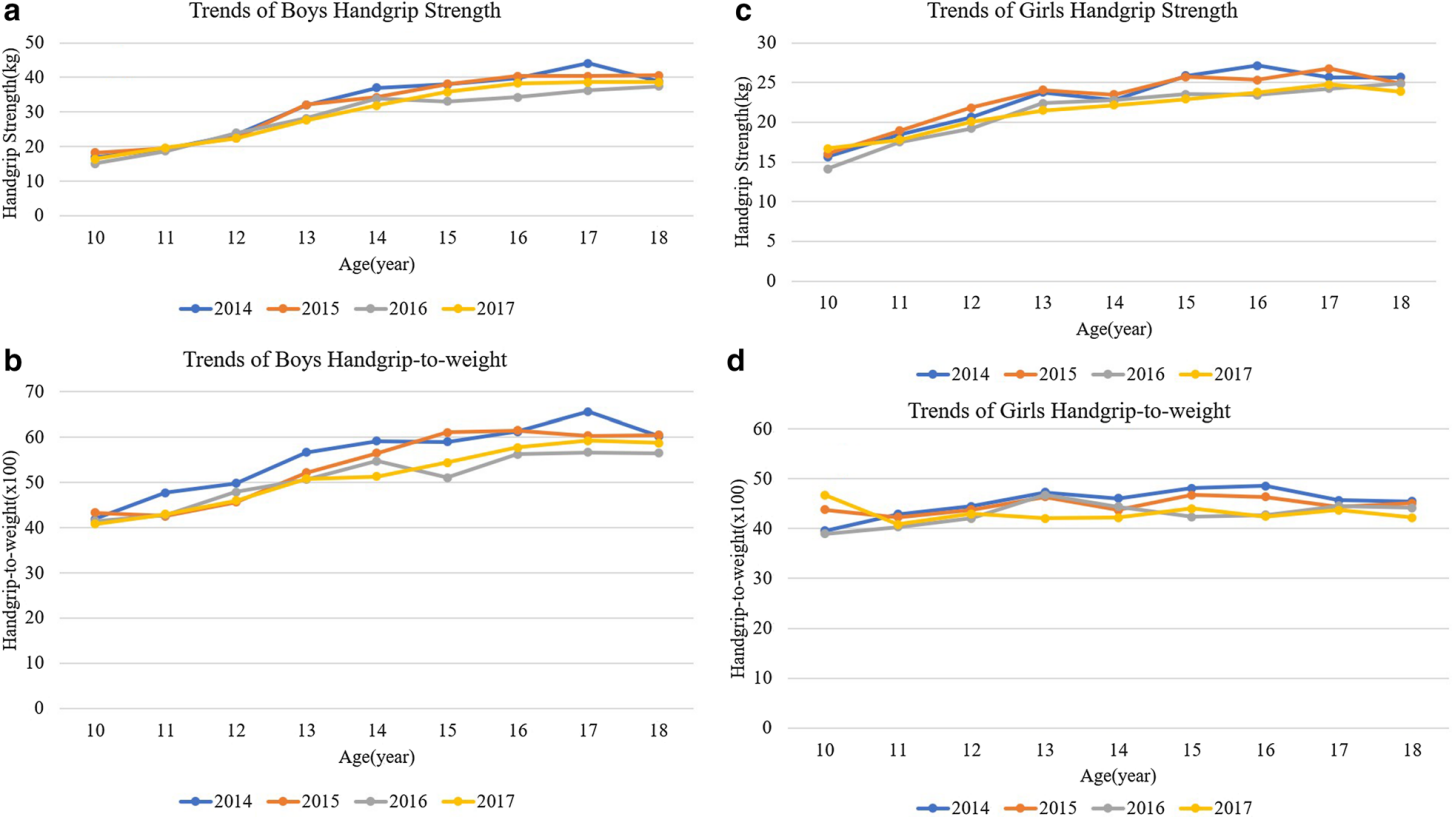
Trends of handgrip strength (**a**) and handgrip-to-weight ratio (×100) (**b**) of boy participants. Trends of handgrip strength (**c**) and handgrip-to-weight ratio (×100) (**d**) among girl participants

**Fig. 2:**
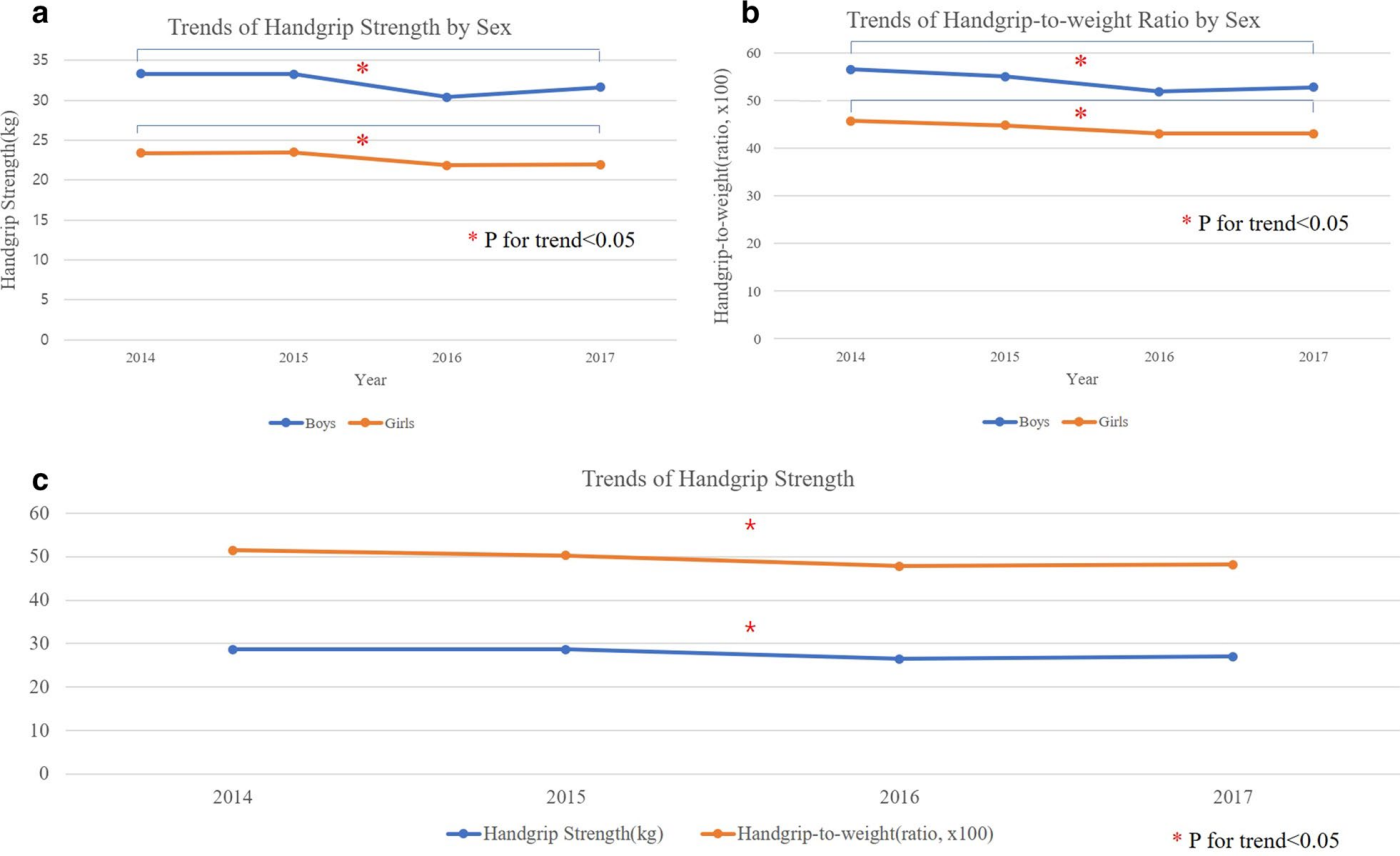
Trends of handgrip strength (**a**) and handgrip-to-weight ratio (×100) (**b**) by sex. Trends of handgrip strength and handgrip-to-weight ratio among adolescents (boys and girls) over the years 2014 to 2017 (**c**). The HG and HGtW significantly decreased from 2014 to 2017 (*P for trend < 0.05) P for trend was analyzed by linear regression with applying weight factors of survey design

The trends in HG and HGtW among the boys are shown by year and age (Fig. 1a, b). The overall (age 10 to 18) trends of boy’s HG and HGtW ratio is decreasing over 2014 to 2017. HG and HGtW ratio decreased from 33.3 kg to 31.6 kg and from 56.5 to 52.8, respectively (P for trend < 0.05) (Fig. 2a). The HG significantly decreased among 13-, 14-, 15-, and 17-year-old boys. Furthermore, HGtW ratio decreased in 13-, 14-, 15-, 16-, and 17-year-old boys (P for trend < 0.05) (Additional file 1: Tables S1, S2).

#### Trends of handgrip strength among girls from 2014 to 2017

The trends of HG and HGtW ratio among the girls are shown by year and age (Fig. 1c, d). The results showed a similar trend for HG and HGtW ratio among the girls as with the boys over the years (P for trend < 0.05). The HG decreased from 23.38 kg to 21.92 kg and HGtW ratio decreased from 45.7 to 43.1 (Fig. 2b). The HG decreased significantly in 13-, 14-, 15-, 16-, and 17-year-old girls. Furthermore, HGtW ratio decreased in 13-, and 16-year-old girls (P for trend < 0.05) (Additional file 1: Tables S3, S4).

#### Trends of handgrip strength among adolescents from 2014 to 2017

The trends of HG and HGtW ratio among the overall adolescents (boys and girls) decreased over the years significantly, from 28.7 kg to 27.0 kg and from 51.5 to 48.2, respectively (P for trend < 0.05) (Fig. 2c) (Additional file 1: Table S5).

### Discussion

Anthropometric measurements, such as height and weight are basic but important examinations for children and adolescents. Measuring height and weight may help in screening several diseases such as nonalcoholic fatty liver disease (NAFLD), metabolic syndrome. These non-invasive methods of obtaining information can be a good evaluation method for patients as well as pediatricians, especially for children and adolescents in whom we need to minimize invasive screening. However, even though various study shows importance of HG, it is still not used much compared to height, body weight, BMI, SBP, DBP. Some studies have demonstrated a relationship between muscle mass and current health status [2, 15]. Cohen et al. showed the importance of muscle strength being associated with metabolic risk factors in children [21]. Moreover, Grontved et al. showed that adolescents’ muscle strength is associated with cardiovascular risk in young adulthood [22]. In addition to the above-mentioned metabolic syndrome-related diseases, many studies have demonstrated HG’s relationship with functional and psychological health as well as quality of life [12, 13, 17, 23]. This study showed a significant decrease in HG and HGtW ratio among adolescent participants over the years from 2014 to 2017. The decrease in HG and HGtW indicates muscle strength has been decreased. Moreover, it might indicate that some of the disorders mentioned above are increasing.

A recent study of handgrip cutoffs performed in children and adolescents in Colombia reported that the lower the HGtW ratio, the higher the likelihood of cardiometabolic risk [24]. Compared with the HG cut-off presented in a previous study, the HGtW ratio among boys and girls aged 10–12 years are higher (0.376 for boys and 0.359 for girls). In the present study, from 2014 to 2017, boys over 13 years of age showed an HGtW higher than the cut-off value (0.447 for boys) in a previous study. Girls’ HGtW, however, was higher than the cut-off value (0.440 for girls) in year 2014, but the HGtW was found to be low in 2017. HGtW of Korean adolescents are higher than the cut-off value seen in the Colombia study, but it is declining over the years. Specifically, in 2017, HGtW of girls over 13 years of age were lower than the cut-off value.

Other variables except WtHR and cholesterol did not show significant trends over years, but HG and HGtW are decreasing over years and it can be affected by various variables as mentioned above. Therefore, attention is required even though there has been no significant change in blood pressure and laboratory test results in Korean adolescents. Currently, the situation in Korea does not seem to be irrelevant. The health status can be easily inferred by HG and HGtW, and this study implies the risk of health problems of Korean adolescents have begun to develop.

To summarize, the results of the present study showed a decrease in HG and HGtW in Korean adolescents, which might be indicative of a problem in overall health status among Korean adolescents. Decrease in HG and HGtW might be related to decreased physical activity levels or poorer dietary intakes. To our knowledge, this is first study to evaluate trends of HG and HGtW in adolescents and to show changes over the years. The overall decrease in HG and HGtW suggests deterioration of the health status among Korean adolescents. Hence, there is a need to review the health of Korean adolescents, and measures should be taken to prevent its deterioration.

## Limitations

This study has a few limitations. First, in the KNHANES, muscle mass was not measured using dual-energy X-ray absorptiometry or imaging studies. Second, there is a lack of information on the cut-off value of HGtW in Korean adolescents. If additional data are collected or cohorts are built in the future, it may be possible to determine cut-off values. Third, missing data were present that could affect the result. Further effort is needed to minimize the missing data because it represents the whole Korean adolescent. Fourth, puberty can affect the result of handgrip strength, however, tanner staging data is missing in KNHANES data. Fifth, physical activity, dietary intake or socioeconomic status can affect handgrip strength, but these variables were excluded in analysis [25]. However, despite these limitations, this is the first large-scale study to evaluate trends of handgrip strength in Korean adolescents.

## Supplementary information

**Additional file 1: Table S1.** Handgrip strength in boys. **Table S2.** Handgrip strength to weight in boys. **Table S3.** Handgrip strength in girls. **Table S4.** Handgrip strength to weight in girls. **Table S5.** Handgrip strength and handgrip strength to weight in adolescents.

## Data Availability

The KNHANES data used in this study is available in http://knhanes.cdc.go.kr.

## Abbreviations

KNHANES: Korean adolescents using data from the Korea National Health and Nutrition Examination Survey
NAFLD: Nonalcoholic fatty liver disease
SBP: Systolic blood pressure
DBP: Diastolic blood pressure
BMI: Body mass index
WtHR: Waist-to-height ratio
AST: Aspartate aminotransferase
ALT: Alanine aminotransferase
TG: Triglyceride
HDL: High-density lipoprotein
HG: Handgrip strength
HGtW: Handgrip-to-weight
