# Supplementary material for: Trends in measures of handgrip strength from 2014 to 2017 among Korean adolescents using the Korean National Health and Nutrition Examination Survey Data
Source: BMC Res Notes. 2020 Jun 30;13:307. doi: 10.1186/s13104-020-05112-3 (PMC7329499; doi:10.1186/s13104-020-05112-3)
Supplement: Supplementary file 1 — Additional file 1: Table S1. Handgrip strength in boys. Table S2. Handgrip strength to weight in boys. Table S3. Handgrip strength in girls. Table S4. Handgrip strength to weight in girls. Table S5. Handgrip strength and handgrip strength to weight in adolescents. [file 13104_2020_5112_MOESM1_ESM.docx]

| Age(year) | 2014  (n=259) | 2015  (n=318) | 2016  (n=332) | 2017  (n=318) | *B* coefficient | *P* for trend |
| --- | --- | --- | --- | --- | --- | --- |
| 10  (n=124) | 16.88±0.64 | 18.28±0.67 | 15.07±0.26 | 16.37±0.61 | -0.462 | 0.118 |
| 11  (n=141) | 19.31±0.52 | 19.49±0.63 | 18.72±0.47 | 19.64±0.89 | -0.002 | 0.994 |
| 12  (n=126) | 23.49±1.02 | 22.46±0.83 | 23.92±1.15 | 22.35±1.07 | -0.206 | 0.660 |
| 13  (n=165) | 32.00±1.33 | 32.05±1.01 | 28.12±0.95 | 27.58±1.06 | -1.759 | 0.001 |
| 14  (n=138) | 36.94±1.53 | 34.28±1.19 | 33.80±1.36 | 31.81±1.18 | -1.592 | 0.016 |
| 15  (n=144) | 37.93±0.85 | 38.07±0.97 | 33.11±1.03 | 35.73±1.29 | -1.172 | 0.021 |
| 16  (n=130) | 39.69±0.85 | 40.35±1.52 | 34.30±1.23 | 38.33±1.13 | -0.978 | 0.055 |
| 17  (n=129) | 44.07±1.33 | 40.31±1.62 | 36.18±0.94 | 38.57±1.07 | -1.858 | 0.001 |
| 18  (n=130) | 38.78±1.98 | 40.61±0.59 | 37.37±1.18 | 38.65±1.23 | -0.491 | 0.443 |
| Total | 33.32±0.81 | 33.24±0.68 | 30.40±0.57 | 31.60±0.61 | -0.084 | 0.012 |

Table S1. Handgrip strength in boys

| Age(year) | 2014  (n=259) | 2015  (n=318) | 2016  (n=332) | 2017  (n=318) | *B* coefficient | *P* for trend |
| --- | --- | --- | --- | --- | --- | --- |
| 10  (n=124) | 41.93±1.44 | 43.35±1.22 | 41.50±1.30 | 40.83±1.24 | -0.525 | 0.381 |
| 11  (n=141) | 47.79±1.59 | 42.60±2.06 | 42.86±1.12 | 42.99±2.18 | -1.499 | 0.083 |
| 12  (n=126) | 49.82±1.99 | 45.69±1.38 | 47.88±2.27 | 46.01±1.46 | -0.882 | 0.269 |
| 13  (n=165) | 56.72±1.85 | 52.20±1.37 | 50.66±1.68 | 50.88±1.55 | -1.812 | 0.017 |
| 14  (n=138) | 59.22±2.27 | 56.53±2.01 | 54.73±2.82 | 51.37±1.96 | -2.501 | 0.015 |
| 15  (n=144) | 58.98±1.56 | 61.08±1.61 | 51.12±1.59 | 54.43±1.45 | -2.315 | 0.002 |
| 16  (n=130) | 61.24±1.49 | 61.56±2.11 | 56.23±2.54 | 57.77±1.60 | -1.548 | 0.043 |
| 17  (n=129) | 65.61±2.00 | 60.39±2.04 | 56.70±1.88 | 59.27±1.79 | -2.017 | 0.021 |
| 18  (n=130) | 60.23±3.24 | 60.52±1.23 | 56.53±1.74 | 58.77±1.79 | -0.876 | 0.393 |
| Total | 56.54±0.89 | 55.05±0.87 | 51.93±0.87 | 52.82±0.70 | -1.424 | <0.001 |

Table S2. Handgrip strength to weight in boys

| Age(year) | 2014  (n=224) | 2015  (n=256) | 2016  (n=302) | 2017  (n=295) | *B* coefficient | *P* for trend |
| --- | --- | --- | --- | --- | --- | --- |
| 10  (n=105) | 15.66±1.64 | 16.03±0.79 | 14.14±0.58 | 16.67±1.70 | -0.525 | 0.381 |
| 11  (n=115) | 18.41±0.72 | 18.91±1.03 | 17.51±0.56 | 17.76±0.69 | -1.499 | 0.083 |
| 12  (n=120) | 20.65±0.83 | 21.79±0.91 | 19.19±0.71 | 20.06±0.74 | -0.882 | 0.269 |
| 13  (n=133) | 23.77±0.91 | 24.03±1.05 | 22.36±0.65 | 21.49±0.67 | -1.812 | 0.017 |
| 14  (n=127) | 22.76±0.63 | 23.46±0.76 | 22.78±0.62 | 22.16±0.98 | -2.501 | 0.015 |
| 15  (n=118) | 25.87±1.36 | 25.71±0.86 | 23.53±0.75 | 22.91±0.67 | -2.315 | 0.002 |
| 16  (n=133) | 27.12±0.67 | 25.34±0.71 | 23.41±0.72 | 23.78±0.67 | -1.548 | 0.043 |
| 17  (n=129) | 25.68±1.08 | 26.73±0.67 | 24.23±0.74 | 24.75±0.56 | -2.017 | 0.021 |
| 18  (n=97) | 25.65±1.25 | 24.92±0.88 | 24.83±0.79 | 23.86±0.90 | -0.543 | 0.276 |
| Total | 23.38±0.48 | 23.50±0.34 | 21.84±0.33 | 21.92±0.31 | -0.607 | 0.001 |

Table S3. Handgrip strength in girls

| Age(year) | 2014  (n=224) | 2015  (n=256) | 2016  (n=302) | 2017  (n=295) | *B* coefficient | *P* for trend |
| --- | --- | --- | --- | --- | --- | --- |
| 10  (n=105) | 39.52±1.29 | 43.81±1.88 | 38.98±1.68 | 46.74±4.70 | 1.798 | 0.261 |
| 11  (n=115) | 42.91±1.33 | 42.26±1.29 | 40.31±1.47 | 40.83±1.24 | -0.817 | 0.167 |
| 12  (n=120) | 44.51±1.55 | 43.80±1.51 | 42.09±1.33 | 42.94±1.57 | -0.635 | 0.365 |
| 13  (n=133) | 47.27±1.59 | 46.35±1.71 | 46.76±1.53 | 42.02±1.42 | -1.556 | 0.020 |
| 14  (n=127) | 46.08±1.17 | 43.75±1.25 | 44.37±1.44 | 42.23±1.49 | -1.070 | 0.070 |
| 15  (n=118) | 48.09±3.13 | 46.74±1.80 | 42.37±1.24 | 43.99±1.33 | -1.628 | 0.096 |
| 16  (n=133) | 48.57±1.39 | 46.33±1.47 | 42.76±1.55 | 42.45±1.20 | -2.180 | 0.001 |
| 17  (n=129) | 45.67±2.06 | 44.33±1.07 | 44.55±1.40 | 43.69±1.53 | -0.570 | 0.471 |
| 18  (n=97) | 45.45±1.80 | 45.07±2.72 | 44.16±1.33 | 42.18±1.72 | -1.089 | 0.201 |
| Total | 45.73±0.71 | 44.86±0.64 | 43.07±0.53 | 43.05±0.66 | -0.978 | 0.001 |

Table S4. Handgrip strength to weight in girls

Table S5. Handgrip strength and handgrip strength to weight in adolescents

|  | 2014  (n=483) | 2015  (n=574) | 2016  (n=634) | 2017  (n=613) | *B* coefficient | *P* for trend |
| --- | --- | --- | --- | --- | --- | --- |
| Handgrip strength | 28.67±0.64 | 28.67±0.50 | 26.43±0.43 | 27.00±0.42 | -0.724 | 0.003 |
| Handgrip to weight | 51.48±0.73 | 50.26±0.64 | 47.83±0.63 | 48.18±0.52 | -1.228 | <0.001 |
